# Pre-pregnancy body mass index and glycated-hemoglobin with the risk of metabolic diseases in gestational diabetes: a prospective cohort study

**DOI:** 10.3389/fendo.2023.1238873

**Published:** 2023-09-28

**Authors:** Xinyue Wang, Simin Zhang, Wenlu Yu, Guohua Li, Jinglin Li, Jing Ji, Yang Mi, Xiaoqin Luo

**Affiliations:** ^1^ Department of Nutrition and Food Safety, School of Public Health, Xi’an Jiaotong University, Xi’an, China; ^2^ Department of Obstetrics and Gynecology, Northwest Women’s and Children’s Hospital, Xi’an, China

**Keywords:** pre-pregnancy body mass index, high glycated hemoglobin, gestational metabolic diseases, gestational diabetes mellitus, gestational hypertension

## Abstract

**Background:**

Metabolic diseases during pregnancy result in negative consequences for mothers. Pre-pregnancy body mass index (BMI) and late-pregnancy glycated-hemoglobin (HbA1c) are most important factors independently affecting the risk of gestational diabetes mellitus (GDM). However how both affect the combined risk of other metabolic diseases in women with GDM is unclear. The study aims to investigate the influence of pre-pregnancy BMI and pregnancy glycemic levels on other gestational metabolic diseases in women with GDM.

**Methods:**

Pregnancies with GDM from January 2015 to December 2018 in the Xi’an longitudinal mother-child cohort study (XAMC) were retrospectively enrolled. Those without other metabolic diseases by the time of oral glucose tolerance test (OGTT) detection were finally recruited and divided into four groups by pre-pregnancy BMI (Underweight <18.5kg/m^2^; Normal weight 18.5-23.9 kg/m^2^; Overweight 24.0-27.9 kg/m^2^; Obesity ≥28.0 kg/m^2^, respectively) or two groups by HbA1c in late pregnancy (normal HbA1c<5.7%; high HbA1c≥5.7%). Multivariate logistic regression analysis was used to identify risk factors. Interaction between pre-pregnancy BMI (reference group 18.5-23.9 kg/m^2^) and HbA1c (reference group <5.7%) was determined using strata-specific analysis.

**Results:**

A total of 8928 subjects with GDM were included, 16.2% of which had a composite of metabolic diseases. The pre-pregnancy overweight and obesity, compared with normal BMI, were linked to the elevated risk of the composite of metabolic diseases, particularly pre-eclampsia (both *P* <0.001) and gestational hypertension (both *P* <0.001). Meanwhile, patients with high HbA1c had an obvious higher risk of pre-eclampsia (*P*< 0.001) and gestational hypertension (*P*= 0.005) compared to those with normal HbA1c. In addition, there were significant interactions between pre-pregnancy BMI and HbA1c (*P*< 0.001). The *OR* of pre-pregnancy BMI≥ 28 kg/m^2^ and HbA1c≥ 5.7% was 4.46 (95% CI: 2.85, 6.99; *P*< 0.001). The risk of other metabolic diseases, except for pre-eclampsia (*P*= 0.003), was comparable between the two groups of patients with different HbA1c levels at normal pre-pregnancy BMI group. However, that was remarkably elevated in obese patients (*P*= 0.004), particularly the risk of gestational hypertension (*P*= 0.004).

**Conclusion:**

Pre-pregnancy overweight/obesity and late-pregnancy high HbA1c increased the risk of other gestational metabolic diseases of women with GDM. Monitoring and controlling late-pregnancy HbA1c was effective in reducing metabolic diseases, particularly in those who were overweight/obese before conception.

## 1 Introduction

Gestational diabetes mellitus (GDM), defined as poor glucose tolerance that develops or first occurs during pregnancy (1), is highly prevalent affecting around 4.4% (2) of pregnancies worldwide and 15% in China (3). It can trigger a series of adverse pregnancy outcomes like cesarean delivery, perinatal mortality and macrosomia (4). Notably, the chronic insulin resistance induced by GDM (5), combined with the physiological changes caused by pregnancy (6), puts women with GDM more likely to develop other metabolic diseases, including gestational hypertension (7) and hypothyroidism (8). Thus, studying the risk factors for other metabolic diseases in patients with GDM is essential.

Over the past decades, the prevalence of overweight and obesity has been rising steadily among all age groups (9). A research reported that of Chinese childbearing women, 25.9% were overweight and 9.2% were obese, higher than that of other Asian countries (10). These were associated with the higher incidence of GDM, gestational hypertension and a lot of adverse pregnant outcomes (11). In addition, one third of pre-pregnancy obese women had higher glycated hemoglobin (HbA1c) levels at delivery (12), which were associated with gestational hypertension, preterm birth, and low birth weight (13). Since the results of oral glucose tolerance test (OGTT) reflects the instantaneous glycemic profile, while the value of HbA1c reflects the average glycemic levels over the past 2 to 3 months, it is potential to make HbA1c a remarkable predictor of gestational complications (14).

However, few researches have focused on the effects of late-pregnancy HbA1c, even less on the combined impact of pre-pregnancy BMI and HbA1c on other metabolic diseases in patients with GDM. Here, we retrospectively enrolled singleton pregnant women with GDM from the Xi’an longitudinal mother-child cohort (XAMC) to address the problem.

## 2 Methods

### 2.1 Study population

**Figure 1:**
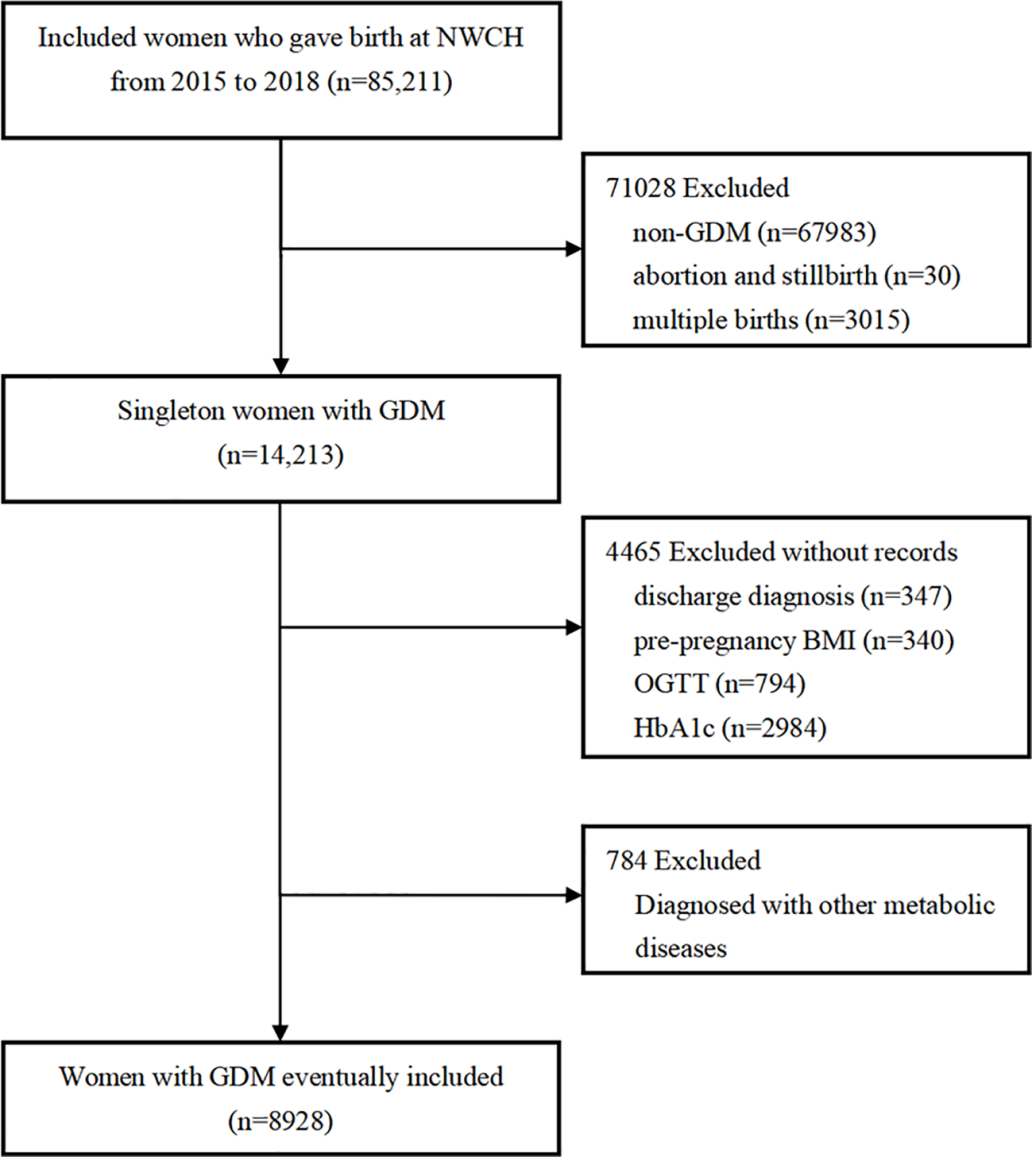
Flow chart of the participants. NWCH, the Northwest Women and Children’s Hospital; BMI, body mass index; OGTT, oral glucose tolerance test; GDM, gestational diabetes mellitus; HbA1c, glycated hemoglobin. Non-GDM included diabetes mellitus complicated pregnancies, and was excluded. Subjects with other gestational metabolic diseases at enrolment were excluded. Other gestational metabolic diseases included: gestational hypertension, pre-eclampsia, subclinical hypothyroidism, hypothyroidism, hyperthyroidism, intrahepatic cholestasis and hashimoto’s thyroiditis.

This study was conducted among the participants of the XAMC. The cohort recruited women from the Northwest Women’s and Children’s Hospital for antenatal care in early pregnancy to examine the impact of early intrauterine exposure on the outcomes of mothers and their child. The specific protocol and baseline information has been described elsewhere (15). Based on the dynamic XAMC, 85211 pregnancies who delivered from January 2015 to December 2018 were enrolled. The eligible subjects were singleton and required to be diagnosed with GDM and had HbA1c data for the last trimester of pregnancy. Additionally, individuals diagnosed with artificial fertilization, other metabolic diseases upon enrollment, multiple pregnancies, abortion or induced labor, non-gestational diabetes and type 1 or 2 diabetes mellitus before pregnancy, other severe diseases like cancer and disease of immune system, or with incomplete or incorrect data were excluded. Finally, 8928 subjects were included in the data analysis. The detailed elimination process is described in **Figure 1**. The Ethics Committee of Xi’an Jiaotong University (XJTU 2016-053) and Northwest Women’s and Children’s Hospital (NWCH 2012-013) approved the study, which was performed according to Helsinki Declaration. All women provided written informed consent.

### 2.2 Definition of GDM

In China, based on the hospital’s antenatal glucose assessment protocol (16), the OGTT was performed in the mid-pregnancy (24-28 weeks). According to the modified criteria of the International Association of Diabetes and Pregnancy Study Groups (12), GDM is diagnosed when at least one value reaches or exceeds any of the following three thresholds in a 75-g OGTT: 5.1 mmol/L for fasting plasma glucose (PG), 10.0 mmol/L for 1-hour PG and 8.5 mmol/L for 2-hour PG.

### 2.3 Definition of pre-pregnancy BMI and late-pregnancy HbA1c

The weight and height information before pregnancy was measured and recorded by a doctor at the first time of antenatal care, usually before 6 weeks of pregnancy (17). Pre-pregnancy BMI was calculated by the pre-pregnancy weight in kilograms divided by the height squared in meters. According to the criteria of the Chinese National Health Commission, subjects were categorized into four weight groups: Underweight, <18.5 kg/m^2^; Normal weight, 18.5-23.9 kg/m^2^; Overweight, 24.0-27.9 kg/m^2^ and Obesity, ≥28.0 kg/m^2^ (18).

Late pregnancy was defined as the last trimester of pregnancy, which starts from the 28th week of pregnancy until delivery. In this study, data on HbA1c were available from the hospital’s Medical Case System. When the late-pregnancy HbA1c value was at or greater than the threshold value of 5.7%, the term “late-pregnancy dysglycemia” was used, as defined previously (16).

### 2.4 Outcomes variables

The primary outcome was the total prevalence of metabolic diseases occurring after the diagnosis of GDM, defined as the presence of at least one of the following outcomes: gestational hypertension, pre-eclampsia, subclinical hypothyroidism, hypothyroidism, hyperthyroidism, intrahepatic cholestasis and Hashimoto’s thyroiditis, termed the “composite of metabolic diseases”. The secondary outcome was the prevalence of each component of the primary outcome described above. Data on disease diagnoses were obtained from the hospital discharge records of subjects. Gestational hypertension was defined as systolic blood pressure (SBP) ≥140 mmHg and/or diastolic blood pressure (DBP) ≥90 mmHg and required at least two blood pressure measurements in the same arm before diagnosis (19). Pre-eclampsia was defined as gestational hypertension with proteinuria (20). Hypothyroidism was defined as increased thyroid stlmulating hormone (TSH) (2.5-10 mIU/L) in conjunction with a decreased free tetraiodothyronine (FT4) or TSH level of more than 10 mIU/L. Subclinical hypothyroidism was defined as elevated TSH (2.5-10 mIU/L) and normal FT4 concentration (21). Hyperthyroidism manifests as TSH< 0.1mIU/L and normal FT4 with laboratory confirmation of the diagnosis (22).

### 2.5 Statistical analysis

The normality of the continuous data distribution was assessed by the Shapiro-Wilk test. As all continuous variables in this study did not conform to normal distributions, they were expressed as medians and quartiles and analyzed by applying the Kruskal-Wallis H test. Categorical variables were described by counts and percentages. When categorical variables met the Cochran hypothesis, the analysis was conducted using a *Chi*-square test, otherwise *Fisher’s* exact test was used. The associations of pre-pregnancy BMI and late HbA1c with specific metabolic diseases were determined by logistic regression of odds ratios (*ORs*) and 95% confidence intervals (*CI*). Based on the results of previous studies, we adjusted for potential confounding, including maternal age, education level, parity, previous cesarean delivery, family history of hypertension and family history of diabetes, and calculated adjusted odds ratios (*aORs*) and 95% *CI*. The interaction term between the pre-pregnancy BMI categories and HbA1c was used to explore the effect of their interaction on metabolic diseases. If the interaction was of statistical significance, strata-specific analysis was then performed. All data were analyzed by SPSS26.0 (Chicago, IL, USA). The figures were generated using GraphPad Prism 8 and R version 4.2.1. All *P* values were two-tailed, with a significance level set at 0.05.

## 3 Results

### 3.1 Baseline characteristics

Participants were at a median and quartile 31 (29, 34) years of age, 24.2% of them were over 35 years old. A majority (89.0%) had high school and above education; over 16% were overweight and 2.6% were obese before conception. Almost 61.4% of mothers were multiparities and 12.6% had a history of cesarean section. As shown in **Table 1**, all the maternal demographic characteristics in different pre-pregnancy BMI categories were varied significantly. Those who were overweight or obese reported having higher prevalence of family history of diabetes or hypertension. In addition, the biochemical indicators of glycolipid metabolism at the late pregnancy showed statistically significant differences in the distribution of pre-pregnancy BMI (all *P* <0.001). When categorized by late-pregnancy HbA1c, one in five of the patients had a higher median HbA1c levels [5.9% (5.7%, 6.0%)] and higher gestational weight gain (GWG) [14.5 (11.0, 17.5) kg] than patients with normal HbA1c levels. The total cholesterol, LDL and triglyceride of the high HbA1c group were markedly higher than those of normal HbA1c group in late pregnancy (*P*= 0.010, 0.031 and 0.018, respectively).

**Table 1:** Maternal demographic and pregnancy characteristics by pre-pregnancy BMI and late-pregnancy HbA1c categories.

Characteristic	Pre-pregnancy Body Mass Index					HbA1c		
	Underweight	Normal weight	Overweight	Obesity	*P-value*	Normal	High	*P-value*
Case (%)	857 (9.6)	6406 (71.8)	1436 (16.1)	229 (2.6)		7067 (79.2)	1861 (20.8)	
Age (%)					<0.001			0.910
<35	736 (85.9)	4809 (75.1)	1039 (72.4)	181 (79.0)		5353 (75.7)	1412 (75.9)	
≥35	121 (14.1)	1597 (24.9)	397 (27.6)	48 (21.0)		1714 (24.3)	449 (24.1)	
Education level (%)					<0.001			0.180
8th grade or less	100 (11.7)	853 (13.3)	245 (17.1)	48 (21.0)		951 (13.8)	271 (14.6)	
High school	612 (71.4)	4517 (70.5)	1018 (73.0)	167 (72.9)		4986 (70.6)	1328 (71.4)	
College and above	140 (16.3)	999 (15.6)	165 (11.5)	11 (4.8)		1067 (15.1)	248 (13.3)	
Parity (%)					<0.001			0.003
Nultiparity	422 (49.2)	2458 (38.4)	480 (33.4)	84 (36.7)		2671(37.8)	773(41.5)	
Multiparities	435 (50.8)	3948 (61.6)	956 (66.6)	145 (63.3)		4396 (62.2)	1088 (58.5)	
Previous cesarean delivery (%)	132 (15.4)	802 (12.5)	169 (11.8)	24 (10.5)	0.047	812 (11.5)	315 (16.9)	<0.001
Family history of diabetes (%)	69 (8.1)	630 (9.8)	166 (11.6)	38 (16.6)	<0.001	665 (9.4)	238 (12.8)	<0.001
Family history of hypertension (%)	107 (12.5)	986 (15.4)	256 (17.8)	54 (23.6)	<0.001	1092 (15.5)	311 (16.7)	0.184
Total GWG (kg)	15 (12.0, 18.0)	14 (10.5, 16.5)	11.5 (9.0, 15.0)	10 (6.3, 14.0)	<0.001	13.0 (10.0, 16.0)	14.5 (11.0, 17.5)	<0.001
Gestational age (weeks)	39 (38, 40)	39 (38, 40)	39 (38, 40)	39 (38, 40)	0.002	39 (38, 40)	39 (38, 40)	0.925
**Biochemical Indicators at the late pregnancy**								
HbA1c (%)	5.3 (5.0, 5.5)	5.3 (5.1 ,5.6)	5.4 (5.1, 5.7)	5.5 (5.2, 5.8)	<0.001	5.2 (5.0, 5.4)	5.9 (5.7, 6.0)	<0.001
Total cholesterol (mmol/l)	5.8 (5.1, 6.6)	5.7 (4.9, 6.5)	5.4 (4.8, 6.2)	5.3 (4.5, 6.0)	<0.001	5.6 (4.9, 6.4)	5.6 (4.8, 6.3)	0.010
HDL (mmol/L)	1.7 (1.5, 1.9)	1.7 (1.5, 1.9)	1.6 (1.4, 1.8)	1.6 (1.4, 1.8)	<0.001	1.7 (1.4, 1.9)	1.6 (1.4, 1.9)	0.176
LDL (mmol/L)	3.0 (2.5, 3.5)	2.8 (2.3, 3.3)	2.7 (2.2, 3.2)	2.6 (2.1, 3.0)	<0.001	2.8 (2.3, 3.3)	2.8 (2.3, 3.3)	0.031
Triglycerides (mmol/L)	2.7 (2.1, 3.4)	3.0 (2.4, 3.9)	3.1 (2.5, 4.1)	3.1 (2.5, 3.8)	<0.001	3.0 (2.4, 3.9)	3.0 (2.4, 4.0)	0.018
**Oral glucose tolerance test (mmol/L)**								
Fasting PG	5.1 (4.7, 5.3)	5.2 (4.9, 5.4)	5.3 (5.1, 5.6)	5.34 (5.14, 5.6)	<0.001	5.2 (4.8, 5.4)	5.3 (5.1, 5.6)	<0.001
1h PG	9.4 (7.9, 10.4)	9.4 (8.1, 10.5)	9.8 (8.4, 10.8)	10.0 (8.9, 10.8)	<0.001	9.4 (6.9, 8.9)	10.0 (8.6, 11.0)	<0.001
2h PG	7.9 (6.8, 9.0)	8.0 (6.9, 9.0)	7.9 (6.9, 9.0)	7.73 (6.8, 8.7)	0.048	7.9 (6.9, 8.9)	8.2 (7.1, 9.2)	<0.001

BMI, body mass index; GWG, gestational weight gain; HDL, high density lipoprotein; LDL, low density lipoprotein; PG, plasma glucose.

Continuous variables are expressed as the median (quartile). Categorical variables are expressed as n (%).

### 3.2 Prevalence of metabolic diseases by pre-pregnancy BMI and late-pregnancy HbA1c

**Figure 2:**
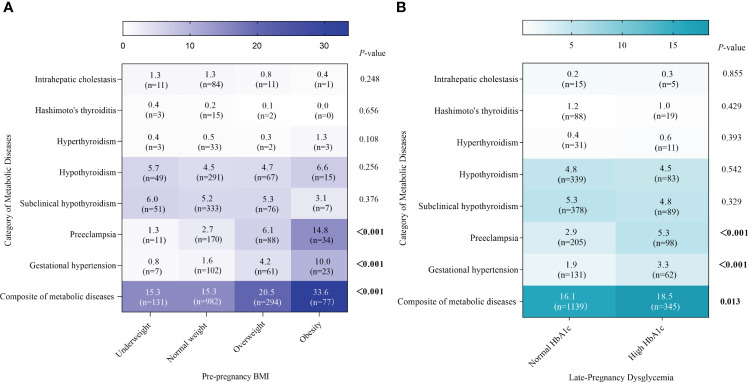
Prevalence of metabolic diseases by pre-pregnancy BMI and late-pregnancy HbA1c. Absolute prevalence of metabolic diseases are indicated by the numerals and shading within the cells, and corresponding frequencies are also shown within the cells. **(A**, **B)** represents the metabolic diseases by BMI groups and HbA1c groups, respectively.

Totally, 16.6% (n= 1484) of GDM women developed metabolic diseases during pregnancy, of which the prevalence was higher in the overweight (20.5%) and obesity groups (33.6%) compared to the normal BMI group (15.3%), respectively. No statistically significant differences were found between the four types group for subclinical hypothyroidism and hypothyroidism (*P*= 0.376, 0.256, respectively). The prevalence of pre-eclampsia and gestational hypertension differed among the BMI groups, with the prevalence increasing progressively with increasing BMI (both *P* <0.001) (**Figure 2A**; **Supplementary Table S1**). Meanwhile, the prevalence of composite of metabolic diseases in the high HbA1c group was significantly higher than in the normal HbA1c group (18.5% & 16.1%, *P*= 0.013), as well as gestational hypertension (3.3% & 1.9%, *P*<0.001), and pre-eclampsia (5.3% & 2.9%, *P*<0.001) (**Figure 2B**; **Supplementary Table S2**).

### 3.3 The influence of pre-pregnancy BMI and late-pregnancy HbA1c on various metabolic diseases

**Figure 3:**
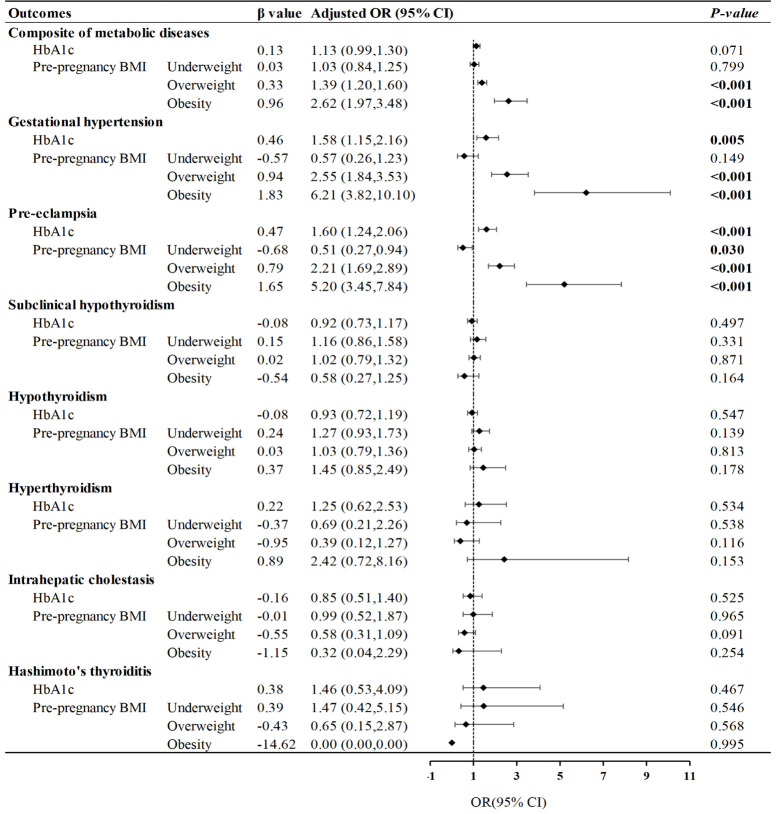
Influence of glycated hemoglobin and pre-pregnancy BMI on metabolic diseases. Forest plot of odds ratios with 95% CIs, for the risk of metabolic diseases according to pre-pregnancy BMI (<18.5; 25.0-29.9; ≥30.0), compared with pre-pregnancy normal weight (BMI: 18.5-24.9), and high HbA1c (≥5.7%), compared with normal HbA1c (<5.7%). The adjusted model was controlled for maternal age, maternal education level, parity, previous cesarean delivery, family history of diabetes and hypertension.

Participants with high HbA1c levels were at higher risk of hypertension and pre-eclampsia, which is demonstrated in **Figure 3** (*P*= 0.004, *P* <0.001, respectively). According to the pre-pregnancy BMI, women with GDM who were overweight or obese had a notable risk of the composite of metabolic diseases, especially gestational hypertension and pre-eclampsia, and the risk of pre-eclampsia was significantly reduced in the underweight group (*P*= 0.023). Meanwhile, we found that the interaction term between pre-pregnancy BMI and late-pregnancy HbA1c had an effect on the primary outcome (*P*< 0.001). The *OR* of pre-pregnancy BMI≥ 28 kg/m^2^ and HbA1c≥ 5.7% was 4.46 (95% CI: 2.85, 6.99; *P*< 0.001) (**Supplementary Table S3**).

### 3.4 The effect of HbA1c on metabolic diseases in different groups of pre-pregnancy BMI

We further evaluated the effect of pre-pregnancy BMI on GDM stratified by the pre-pregnancy BMI (**Table 2**). In the categories of underweight and overweight, no significant differences were found in the risk of primary and secondary outcomes between normal and high HbA1c groups. However, in the category of obesity, the risk of the composite of metabolic diseases was sharply increased in the high HbA1c group (46.2% & 27.2%, *P*= 0.004), along with gestational hypertension (17.9% & 6.0%, *P*= 0.004). In addition, in the category of normal weight, the prevalence of pre-eclampsia in the high HbA1c group was also elevated compared to the normal HbA1c group (3.9% & 2.4%, *P*= 0.003).

**Table 2:** Effect of HbA1c on metabolic diseases in different groups of pre-pregnancy BMI.

	Composite of metabolic diseases (%)	Gestational hypertension (%)	Pre-eclampsia (%)	Subclinical hypothyroidism (%)	Hypothyroidism (%)	Hyperthyroidism (%)	Intrahepatic cholestasis (%)	Hashimoto’s thyroiditis (%)
Underweight (n= 857)								
Normal HbA1c	114 (15.1)	7 (1.0)	9 (1.2)	45 (6.1)	42 (5.7)	2 (0.3)	7 (0.9)	3 (0.4)
High HbA1c	17 (14.9)	0	2 (1.8)	6 (5.3)	7 (6.1)	1 (0.9)	4 (3.5)	0
*P-value*	0.905	/	0.974	0.739	0.835	0.349	0.690	/
Normal weight (n= 6406)								
Normal HbA1c	778 (15.1)	76 (1.5)	121 (2.4)	271 (5.3)	237 (4.6)	26 (0.5)	70 (1.4)	11 (0.2)
High HbA1c	204 (16.1)	26 (2.1)	49 (3.9)	62 (4.9)	54 (4.3)	7 (0.6)	14 (1.1)	4 (0.3)
*P-value*	0.387	0.143	**0.003**	0.590	0.597	0.834	0.473	0.728
Overweight (n= 1436)								
Normal HbA1c	206 (19.9)	39 (3.8)	57 (5.5)	56 (5.4)	52 (5.0)	3 (0.3)	10 (1.0)	1 (0.1)
High HbA1c	88 (21.8)	22 (5.5)	31 (7.7)	20 (5.0)	15 (3.7)	0	1 (0.2)	1 (0.2)
*P-value*	0.424	0.155	0.123	0.727	0.290	/	0.285	0.483
Obesity (n= 229)								
Normal HbA1c	41 (27.2)	9 (6.0)	18 (11.9)	6 (4.0)	8 (5.3)	0	1 (0.7)	0
High HbA1c	36 (46.2)	14 (17.9)	16 (20.5)	1 (1.4)	7 (9.1)	3 (1.3)	0	0
*P-value*	**0.004**	**0.004**	0.083	0.474	0.287	/	/	/

Statistically significant values are bolded for p < 0.05.

## 4 Discussion

Our present findings extended previous reports linking GDM, pre-pregnancy BMI and HbA1c with other gestational metabolic diseases. We found that both the higher pre-pregnancy BMI and late-pregnancy HbA1c increased the risk of pre-eclampsia and gestational hypertension in women with GDM. Moreover, better control of glucose metabolism in late pregnancy, which in terms of late normal HbA1c, may significantly decrease the risk of those metabolic diseases, especially in GDM women who were obese before conception.

GDM is one of the most common complications of pregnancy and is characterized by impaired glucose metabolism (23). In this study, 16.6% of women with GDM developed other metabolic diseases, with 5.5% suffering from pre-eclampsia or gestational hypertension, and 10.0% from subclinical hypothyroidism or hypothyroidism. In contrast, in the general population of women, the prevalence of gestational hypertension and pre-eclampsia were about 4.0% and 2.1%, respectively (24), and subclinical hypothyroidism or hypothyroidism was 4.7% (25), all of which were lower than the risks in this study of women with GDM. Meanwhile, the reported risk of gestational hypertension in non-GDM was significantly lower than in GDM (2.5% & 6.8%) (26). Women with GDM may be at high threat for other metabolic diseases (24, 25, 27). Gestational metabolic diseases can produce adverse short- and long-term impairments in the mother and child, such as kidney diseases and child neurodevelopmental disorders (25, 28). More importantly, possible synergistic effects between metabolic diseases may further contribute to the development of serious diseases. Evidence demonstrated that the coexistence of gestational hypertension and GDM increases the risk of cardiovascular disease (29). More researches are needed to unearth the underlying mechanisms of the interactions between GDM and other gestational metabolic diseases. Focusing on the risk of other metabolic diseases in women with GDM and targeting interventions for those at risk for GDM may be of great value.

Previously, a cohort study found that pre-pregnancy obesity was a powerful risk factor for pregnancy complications such as pre-eclampsia and gestational diabetes, to a greater extent than overweight or excessive gestational weight gain (30). Meng Li and et al. (31) further pointed out that higher values of pre-pregnancy BMI can induce GDM to complicate pre-eclampsia, gestational hypertension, preterm delivery and macrosomia. Consistently, we also observed that after adjusting for confounding factors, the prevalence of composite of metabolic diseases remained significantly higher in the overweight and obesity groups than in the normal weight group, particularly pre-eclampsia and gestational hypertension. This highlights that being overweight and obese before pregnancy is not only an independent risk factor for GDM, but also puts women with GDM at increased risk of comorbid other metabolic diseases, especially the disorders of blood pressure.

The pathophysiological mechanisms underlying the links between pre-pregnancy BMI and blood pressure during pregnancy have not been fully elucidated. Being overweight and obese before pregnancy can lead to inflammation, hyperinsulinemia and insulin resistance, further disturbing autonomic dysfunction (32). Overweight/obesity may also elicit disturbances in bioactive compounds, such as lipids, leptin function and adipokines (20). In the current analysis, there were also differences in the results for blood lipids between the BMI groups. The levels of triglyceride were generally high (median >2.7 mmol/L) and were statistically different between the groups. The overweight and obese groups had lower levels of HDL than the normal weight group. The association between hypertriglyceridemia and preeclampsia in pregnancy was previously reported that low levels of HDL were relevant to preeclampsia, but not LDL (33). Triglycerides accumulate in the lining cells of the uterine spiral arteries, resulting in decreased prostacyclin production and may lead to endothelial dysfunction and increased oxidative stress (34). Whether the prevalence of gestational metabolic diseases in pre-pregnancy obese/overweight women would be increased by some degree of lipid alteration remains to conjecture, but appropriate weight management before pregnancy is essential. For those at the high risk of GDM who were overweight/obese before pregnancy, blood pressure and lipid changes should be monitored dynamically during pregnancy to prevent the development of gestational hypertension.

Compared to OGTT for transient measurements, HbA1c represents the average glycemia level over the previous 8-12 weeks and is characterized by being easy to test and unaffected by short-term fluctuations in blood (14). Of note, despite appropriate treatments being given to patients with GDM, they may still have higher late-pregnancy HbA1c levels than pregnancies without GDM (12). Hyperglycemia positively associated with adverse pregnancy outcomes (12). Attention should be paid to the importance of HbA1c as an objective biochemical indicator of glycemic control in women with GDM (35). A recent study revealed that HbA1c ≥5.7% during pregnancy indicated impaired β-cell function and pathophysiological dysfunction of glucose disposal (36). Late-pregnancy HbA1c at or above 5.7% in obese non-GDM pregnancies posed long-term health risks to the offspring and mother (16, 37). When the cut-off value for HbA1c in this study was set at 5.7%, we also found that high HbA1c was an independent risk factor for pre-eclampsia and gestational hypertension in women with GDM. Couples of studies emphasized the important role of HbA1c in the risk of pre-eclampsia. Although there is scarce epidemiological evidence on late-pregnancy HbA1c, the available studies generally supported our results. A large population-based study indicated that the risk of pre-eclampsia increased with elevated mid-pregnancy HbA1c (14). Meanwhile, Lynn P et al. reported on the association of HbA1c measured at 28 weeks as a continuous variable with pre-eclampsia (38). Holmes et al. provided the first evidence that HbA1c <6.1% in late pregnancy reduced the risk of pre-eclampsia in type 1 diabetic women (39). Although the mechanism of this association is not fully clear, some evidence showed that elevated HbA1c may induce endothelial dysfunction by generating superoxide anions that interfere with nitric oxide mediated response (40). Endothelial dysfunction may perturb vascular biomarkers including P-selectin, E-selectin, intercellular adhesion molecules and vascular cell adhesion molecules further impairing the vasculature (41), and thus HbA1c may be associated with hypertension. Our data reinforced previous scientific evidence. Notably, among the various risk factors and mechanisms for pre-eclampsia and gestational hypertension, poor glycemic control remains one of the most easily monitored and treated risk factors (39).

There is an important role of weight management prior to pregnancy in reducing adverse gestational metabolic diseases (42). Meanwhile, evidence suggested that the linkage between HbA1c and adverse pregnancy outcomes differed with pre-pregnancy BMI and GWG levels (43). Indicators of BMI combined with HbA1c can help to assess the prognosis of women with GDM. Given that, we further investigated whether pre-pregnancy BMI interacts with late-pregnancy HbA1c on the risk of metabolic diseases by stratifying the pre-pregnancy BMI. The results showed that the proportion of high late-pregnancy HbA1c gradually increased with elevated pre-pregnancy BMI. Notably, in the obese group, glycemia within the optimal range significantly reduced the risk of metabolic diseases, especially gestational hypertension, despite the diagnosis of GDM. In addition, the high HbA1c group was more likely to suffer from pre-eclampsia even if their pre-pregnancy BMI was normal. Therefore, we recommend that GDM women who have excessive pre-pregnancy BMI should be aware of gestational hypertension. It is advisable to use HbA1c as a clinical indicator to monitor glycemia in the last trimester of pregnancy, to assess the impact of GDM treatment timely, and to adjust the therapy to minimize the long-term hazards caused by metabolic diseases.

There are several advantages of this study. To begin with, the data derived from a large population makes the results more convincing. In addition, an accurate experimental design and data collection was conducted, with adjustments for known or potential confounders. Furthermore, we paid particular attention to the interaction of HbA1c and pre-pregnancy BMI on gestational metabolic diseases, with implications for the clinically appropriate management of women with GDM.

However, our study has some limitations. Firstly, we didn’t have information on the exact gestational age at which BMI and HbA1c were recorded. Secondly, it is a regional study with all participants from Xi’an. Data from a single center may lack representation of the entire pregnancy population and selection bias is inevitable. Thirdly, although comprehensive covariates were included in this study, some potential confounders such as pregnancy lifestyle may modify the association of pre-pregnancy BMI and HbA1c with gestational metabolic diseases, inducing confounding bias. Last but not least, this study has proposed an effect of pre-pregnancy BMI and HbA1c on metabolic diseases, but has not yet explored the specific mechanisms that produce this result. Therefore, further studies will be needed to confirm this relationship.

## 5 Conclusions

In conclusion, our results suggested that women should be reminded to keep their BMI at an optimal range when planning for pregnancy to reduce the risk of gestational metabolic diseases. Continuous monitoring of HbA1c is necessary to manage therapeutic effects in women with GDM, especially in the last trimester of pregnancy. Tailored BMI advice, and measures to control glycemia in late pregnancy appear to be an appropriate intervention for closer preventive follow-up of metabolic diseases.

## Data availability statement

The original contributions presented in the study are included in the article/**Supplementary Material**. Further inquiries can be directed to the corresponding author.

## Ethics statement

The study was approved by both the Research Ethics Committees of NWCH (NWCH2012-013) and Xi’an Jiaotong University (XJTU2016-053). Written informed consent was obtained for all participants.

## Author contributions

XW and SZ contributed equally to this work and share first authorship. XW and SZ conceived the study, analyzed the data, and drafted the original manuscript. WY and JL helped draft the manuscript. GL, JJ, and YM contributed to the review and editing manuscript. XL provided conceptualizations for research and the final revision received. All authors have approved the published version of the manuscript.
