# Supplementary material for: Pre-pregnancy body mass index and glycated-hemoglobin with the risk of metabolic diseases in gestational diabetes: a prospective cohort study
Source: Front Endocrinol (Lausanne). 2023 Sep 28;14:1238873. doi: 10.3389/fendo.2023.1238873 (PMC10569468; doi:10.3389/fendo.2023.1238873)
Supplement: Supplementary file 1 [file Table_1.docx]

**Additional file**

Pre-pregnancy body mass index and glycated-hemoglobin with the risk of metabolic diseases in gestational diabetes: A prospective cohort study

Xinyue Wang^1†^, Simin Zhang^1†^, Wenlu Yu^1^, Guohua Li^1^, Jinglin Li^1^, Jing Ji^1,2^, Yang Mi^2^, Xiaoqin Luo^1*^

Xinyue Wang1^†^**,** Simin Zhang1^†^ contributed equally to this work and share first authorship.

^1^ Department of Nutrition and Food Safety, School of Public Health, Xi’an Jiaotong University, Xi'an, China

^2^ Department of Obstetrics and Gynecology, Northwest Women's and Children's Hospital, Xi'an, China;

*** Correspondence:**Xiaoqin Luo, M.D.

Department of Nutrition and Food Safety, School of Public Health, Xi’an Jiaotong University, Xi'an 710061, China

Telephone: +86-29-82655111 Fax: +86-29-82655111

E-mail: [luoxiaoqin2012@mail.xjtu.edu.cn](mailto:luoxiaoqin2012@mail.xjtu.edu.cn)

**Keywords:** Pre-pregnancy body mass index_1_; High glycated hemoglobin_2_; Gestational metabolic diseases_3_; Gestational diabetes mellitus_4_; Gestational hypertension_5_.

**Total words:** main body 2653 words

**Table S****1 Occurrence of Metabolic diseases by Prepregnancy BMI in GDM**

| *N* (%) | Pre-pregnancy Body Mass Index | | | | P-value |
| --- | --- | --- | --- | --- | --- |
|  | Underweight (BMI<18.5) | Normal weight  (BMI18.5~24.9) | Overweight (BMI25.0~29.9) | Obesity  (BMI≥30.0) |  |
| Composite of metabolic diseases | 131(15.3) | 982(15.3) | 294(20.5) | 77(33.6) | ＜0.001 |
| Gestational hypertension | 7(0.8) | 102(1.6) | 61(4.2) | 23(10.0) | ＜0.001 |
| Preeclampsia | 11(1.3) | 170(2.7) | 88(6.1) | 34(14.8) | ＜0.001 |
| Subclinical hypothyroidism | 51(6.0) | 333(5.2) | 76(5.3) | 7(3.1) | 0.376 |
| Hypothyroidism | 49(5.7) | 291(4.5) | 67(4.7) | 15(6.6) | 0.256 |
| Hyperthyroidism | 3(0.4) | 33(0.5) | 3(0.2) | 3(1.3) | 0.108 |
| Intrahepatic cholestasis | 11(1.3) | 84(1.3) | 11(0.8) | 1(0.4) | 0.248 |
| Hashimoto's thyroiditis | 3(0.4) | 15(0.2) | 2(0.1) | 0(0.0) | 0.656 |

**Table S2 Occurrence of Metabolic diseases by HbA1c in GDM**

| *N* (%) | Normal HbA1c | High HbA1c | P-value |
| --- | --- | --- | --- |
| Composite of metabolic diseases | 1139 (16.1%) | 345 (18.5%) | 0.013 |
| Gestational hypertension | 131 (1.9%) | 62 (3.3%) | ＜0.001 |
| Preeclampsia | 205 (2.9%) | 98 (5.3%) | ＜0.001 |
| Subclinical hypothyroidism | 378 (5.3%) | 89 (4.8%) | 0.329 |
| Hypothyroidism | 339 (4.8%) | 83 (4.5%) | 0.542 |
| Hyperthyroidism | 31 (0.4%) | 11 (0.6%) | 0.393 |
| Intrahepatic cholestasis | 88 (1.2%) | 19 (1.0%) | 0.429 |
| Hashimoto's thyroiditis | 15 (0.2%) | 5 (0.3%) | 0.855 |

**Table S3 The unadjusted OR and 95% CI for the association of pre-pregnancy BMI and HbA1c with outcomes**

|  | Composite of metabolic diseases | Gestational hypertension | Preeclampsia | Subclinical hypothyroidism | Hypothyroidism | Hyperthyroidism | Intrahepatic cholestasis | Hashimoto's thyroiditis |
| --- | --- | --- | --- | --- | --- | --- | --- | --- |
| Pre-pregnancy BMI |  |  | | | | | | |
| Underweight | 1.0 (0.82, 1.22) | 0.51 (0.24, 1.10) | 0.48 (0.26, 0.88)† | 1.15 (0.86, 1.56) | 1.27 (0.93, 1.74) | 0.68 (0.21, 2.22) | 0.98 (0.52, 1.84) | 1.50 (0.43, 5.18) |
| Normal weight | ref. | ref. | ref. | ref. | ref. | ref. | ref. | ref. |
| Overweight | 1.42 (1.23, 164)† | 2.74 (1.99, 3.79)† | 2.40 (1.84, 3.12)† | 1.02 (0.79, 1.32) | 1.03 (0.78, 1.35) | 0.40 (0.12, 1.32) | 0.58 (0.31, 1.10) | 0.59 (0.14, 2.60) |
| Obesity | 2.80 (2.11, 3.71)† | 6.90 (4.30, 11.08)† | 6.40 (4.31, 9.50)† | 0.58 (0.27, 1.23) | 1.47 (0.86, 2.52) | 2.56 (0.78, 8.42) | 0.33 (0.50, 2.38) | 0.002* |
| HbA1c |  |  | | | | | | |
| Normal | ref. | ref. | ref. | ref. | ref. | ref. | ref. | ref. |
| High | 1.18 (1.04,1.35) † | 1.83 (1.34,2.48) † | 1.86 (1.45,2.38) † | 0.89 (0.70,1.13) | 0.93 (0.73,1.18) | 1.35 (0.68, 2.69) | 0.82 (0.50,1.35) | 1.27 (0.46, 3.49) |
| Pre-pregnancy BMI* HbA1c |  |  |  |  |  |  |  |  |
| Underweight* High | 0.91 (0.54,1.53) | 0.000* | 0.57 (0.14,2.31) | 1.00 (0.44, 2.28) | 1.32 (0.61, 2.86) | 1.93 (0.26, 14.19) | 2.93 (1.06, 8.11)† | 0.00* |
| Overweight* High | 1.45 (1.14,1.86) † | 3.01 (1.90,4.75) † | 2.65 (1.80,3.90) † | 0.94 (0.59, 1.48) | 0.78 (0.46, 1.32) | 0.00* | 0.20 (0.03, 1.44) | 1.09 (0.14, 8.15) |
| Obesity* High | 4.46(2.85,6.99) † | 11.39 (6.26,20.75) † | 8.21 (4.67,14.42) † | 0.23 (0.03, 1.68 | 1.99 (0.91, 4.36) | 8.73 (2.64, 28.91)† | 0.00* | 0.00* |

* The sample size is too small to calculate a 95% CI

† There is a significant difference between groups with the same symbol. P values <0.05 were considered statistically significant.
